# Role of surgery in the treatment of pediatric low-grade glioma with various degrees of brain stem involvement

**DOI:** 10.1007/s00381-024-06561-y

**Published:** 2024-08-15

**Authors:** Katalin Nora Lorincz, David Gorodezki, Jens Schittenhelm, Julian Zipfel, Jonas Tellermann, Marcos Tatagiba, Martin Ebinger, Martin Ulrich Schuhmann

**Affiliations:** 1 Section of Pediatric Neurosurgery, University Hospital of Tuebingen, Tuebingen, Germany; 2 Department of Pediatric Oncology, University Children’s Hospital of Tuebingen, Tuebingen, Germany; 3 Department of Neuropathology, Institute of Pathology and Neuropathology, University Hospital of Tuebingen, Tuebingen, Germany; 4 Department of Neurosurgery and Neurotechnology, University Hospital of Tuebingen, Hoppe-Seyler Str. 3, 72076 Tuebingen, Germany

**Keywords:** Pediatric LGG, Brainstem tumor, Neurological outcome, KIA1549::BRAF fusion, Surgical therapy, Tumor growth velocity, 3-Dimensional volumetry

## Abstract

**Objective:**

Posterior fossa pediatric low-grade glioma involving the brainstem and cerebellar peduncles (BS-pLGG) are a subgroup with higher risks at surgery. We retrospectively analyzed the role of surgery in the interdisciplinary armamentarium of treatment options in our institutional series of BS-pLGG with various degrees of brainstem involvement.

**Material and methods:**

We analyzed data of 52 children with BS-pLGG after surgical intervention for clinical/molecular characteristics, neurological outcome, factors influencing recurrence/progression pattern, and tumor volumetric analysis of exclusively surgically treated patients to calculate tumor growth velocity (TGV). Tumors were stratified according to primary tumor origin in four groups: (1) cerebellar peduncle, (2) 4th ventricle, (3) pons, (4) medulla oblongata.

**Results:**

The mean FU was 6.44 years. Overall survival was 98%. The mean PFS was 34.07 months. Two patients had biopsies only. Fifty-two percent of patients underwent remission or remained in stable disease (SD) after initial surgery. Patients with progression underwent further 23 resections, 15 chemotherapies, 4 targeted treatments, and 2 proton radiations. TGV decreased after the 2nd surgery compared to TGV after the 1st surgery (*p* < 0.05). The resection rates were significantly higher in Groups 1 and 2 and lowest in medulla oblongata tumors (Group 4) (*p* < 0.05). More extended resections were achieved in tumors with KIAA1549::BRAF fusion (*p* = 0.021), which mostly occurred in favorable locations (Groups 1 and 2). Thirty-one patients showed postoperatively new neurological deficits. A total of 27/31 improved within 12 months. At the end of FU, 6% had moderate deficits, 52% had mild deficits not affecting activities, and 36% had none. Fifty percent of patients were free of disease or showed remission, 38% were in SD, and 10% showed progression.

**Conclusion:**

The first surgical intervention in BS-pLGG can control disease alone in overall 50% of cases, with rates differing greatly according to location (Groups 1 > 2 > 3 > 4), with acceptable low morbidity. The second look surgery is warranted except in medullary tumors. With multimodality treatments almost 90% of patients can obtain remission or stable disease after > 5 years of follow-up. An integrated multimodal and multidisciplinary approach aiming at minimal safe residual disease, combining surgery, chemo-, targeted therapy, and, as an exception, radiation therapy, is mandatory.

**Supplementary Information:**

The online version contains supplementary material available at 10.1007/s00381-024-06561-y.

## Introduction

In children and adolescents with pediatric low-grade gliomas (pLGG) maximal safe surgical resection is the treatment of choice that yields good long-term stability. Several studies (both single institutional and multicentric) report excellent 10-year overall survival rates and improved progression-free survival after surgical resection [1–3].

Pediatric low-grade gliomas affecting the brainstem, either by infiltration from tumors originating in the cerebellar peduncles or the 4th ventricle or originating within pons or medulla, are a distinctive subgroup of pLGGs within a functionally very sensitive area, carrying potentially a good long-term outcome. However, pLGG are requiring intense interdisciplinary management since surgical therapy is limited by the eloquence of the area, and the long-term survival rates demand that the impact of treatment on the quality of life (QoL) should be minimized [4, 5].

Surgical treatment of this subgroup is possible but challenging due to the eloquence of the brainstem, peduncles, and cranial nerves. Therefore, gross total resection is difficult and often impossible to achieve if long-lasting functional impairments impacting significantly on QoL are to be avoided. Nevertheless, surgery has an important role, since subtotal resection can control further tumor growth as well, while chemotherapy rarely decreases tumor volumes sufficiently, radiation should be avoided in children before completion of puberty, and the emerging role of targeted therapy is not yet defined and not accessible for the majority of patients in a global perspective [6, 7].

Intracranial surgery guided by ultrasound or intraoperative MRI and most importantly extended intraoperative electrophysiological monitoring (IOM) allow maximally safe resection to reduce the tumor to a maximal safe residual tumor volume and limit postoperative disability and morbidity [3].

After preserving eloquent structures of the brainstem at initial surgery, aiming to avoid long-term neurological sequela, further tumor progression either prompts multimodal treatment of BS-pLGG, aiming also to reduce long-term therapy-associated toxicity (adverse effects of chemo/radiotherapy or targeted therapy). A second surgical intervention might be again a treatment option, e.g., postpone radiation therapy in the case of failure of chemotherapy or avoid a long chemotherapy, and its side effects, if there is a chance to further reduce the tumor residual significantly or achieve GTR [8].

It is quite conceivable that tumors originating from outside the brainstem within the cerebellar peduncles or the 4th ventricle and infiltrating the brain stem or, on the other hand, originating within the pons or the medulla might carry a different resectability and prognosis. Furthermore, the location could be associated with different underlying molecular genetic backgrounds.

Apart from the inheritable risk factor for brainstem LGG in case of NF-1, further tumor-related molecular genetic factors like several BRAF-alterations have been discovered in the last decades as prognostic factors [1, 9]. Treatment strategies targeting these molecular genetic alterations successfully are emerging [10–12].

We retrospectively reviewed our patient cohort of surgically treated BS-pLGG regarding clinical presentation, treatment strategy, location and origin of the tumor, the extent of resection, immediate- and long-term neurological outcome, recurrence rates, growth velocity of tumors prior and after surgery, and factors influencing/modifying long term survival of patients in our institution in between 2004 and 2023. The aim is to analyze previous practice and to discuss on the basis of results a possible future role of surgery in these challenging tumors in the light of current options of pediatric neuro-oncology.

The approval was granted by the ethics committee of the Medical Faculty and the University Hospital of Tuebingen (NO 762/2021B02).

## Methods

Patients were retrieved from the pediatric neurosurgery database of our institutional section. We included patients with an age of < 18 years at the time of surgery treated for BS-pLGG according to the 2021 WHO classification between 2006 and 2020.

Clinical data were obtained from the hospital information system concerning clinical presentation, neurological and clinical outcomes during regular outpatient follow-up visits, imaging findings, tumor location, resection rate, neoadjuvant and adjuvant treatments, and histopathological and molecular genetic classification. We reviewed our institutional neuropathology and central pathology reports from the German neuropathology reference centers of the national pediatric brain tumor network, particularly for IDH-2 mutations, BRAF::KIAA1549 fusion, and BRAF-V600E mutation status. BRAF-V600E mutation was tested via pyrosequencing following PCR amplification of exon 15 of the BRAF gene from extracted tumor DNA. BRAF fusion transcript analysis was performed using a brain tumor-specific custom FusionPlex panel (ArcherDX) using mRNA next-generation sequencing.

MRI-based tumor volumetric analysis was performed on pre- and postoperative axial MRI scans. MRI images were taken with 1–3 mm slices at 1.5/3 T MRI. Three-dimensional tumor volumetry was performed using BrainLab Elements (version 3.0, BrainLab, Munich, Germany). A total of 196 MRI scans were analyzed, corresponding to a minimum of 3 MRI scans per eligible patient (3.76 mean MRI scans per patient). The inclusion criteria for the calculation of tumor growth velocity (TGV) (cm^3^/ months) on the basis of computerized volumetric analysis were the availability of a minimum of three sequential MRI scans with identical sequences both pre-and postoperatively enabling comparison as described by Gorodezki in 2022 [6].

Surgical outcome was grouped according to volumetric analysis into GTR (100% of tumor resected) and incomplete resection-IR (< 100% of tumor resected). The group of IR was further divided into subtotal resection (STR, 90–99% of tumor resected) and partial resection (PR, < 90% of tumor resected). Furthermore, the four different location groups were analyzed separately for the extent of resection, disease control, and long-term outcome.

For statistical analysis, we used IBM Statistics SPSS 28.0.0. To test the normality, the Saphiro-Wilk test was performed. For further analysis of data, the Mann–Whitney, Kruskal–Wallis test, median test, paired Mann–Whitney test, and Wilcoxon rank test were applied.

## Results

The total cohort comprised 52 patients. There was almost equal gender distribution — 28 males and 24 females — similar to other reports [13]. The mean age at diagnosis was 8.38 years (min. 1 year, max. 17.5 years), and the time to diagnosis was 5.1 months (min. 0.25 months, max. 48 months). The mean follow-up (regular outpatient visits with control MRI-imaging) was 6.44 years (min.6 months, max. 17 years).

*The histologic classification* showed a predominance of pilocytic astrocytoma (PCA) (*n* = 45), other entities were ganglioglioma (GGL) (*n* = 5), rosette-forming glioneural tumor (RGNT) (*n* = 1) and diffuse astrocytoma (DA), not otherwise specified (NOS) (*n* = 1). No malignant transformation occurred in our cohort.

### Groups

**Fig. 1:**
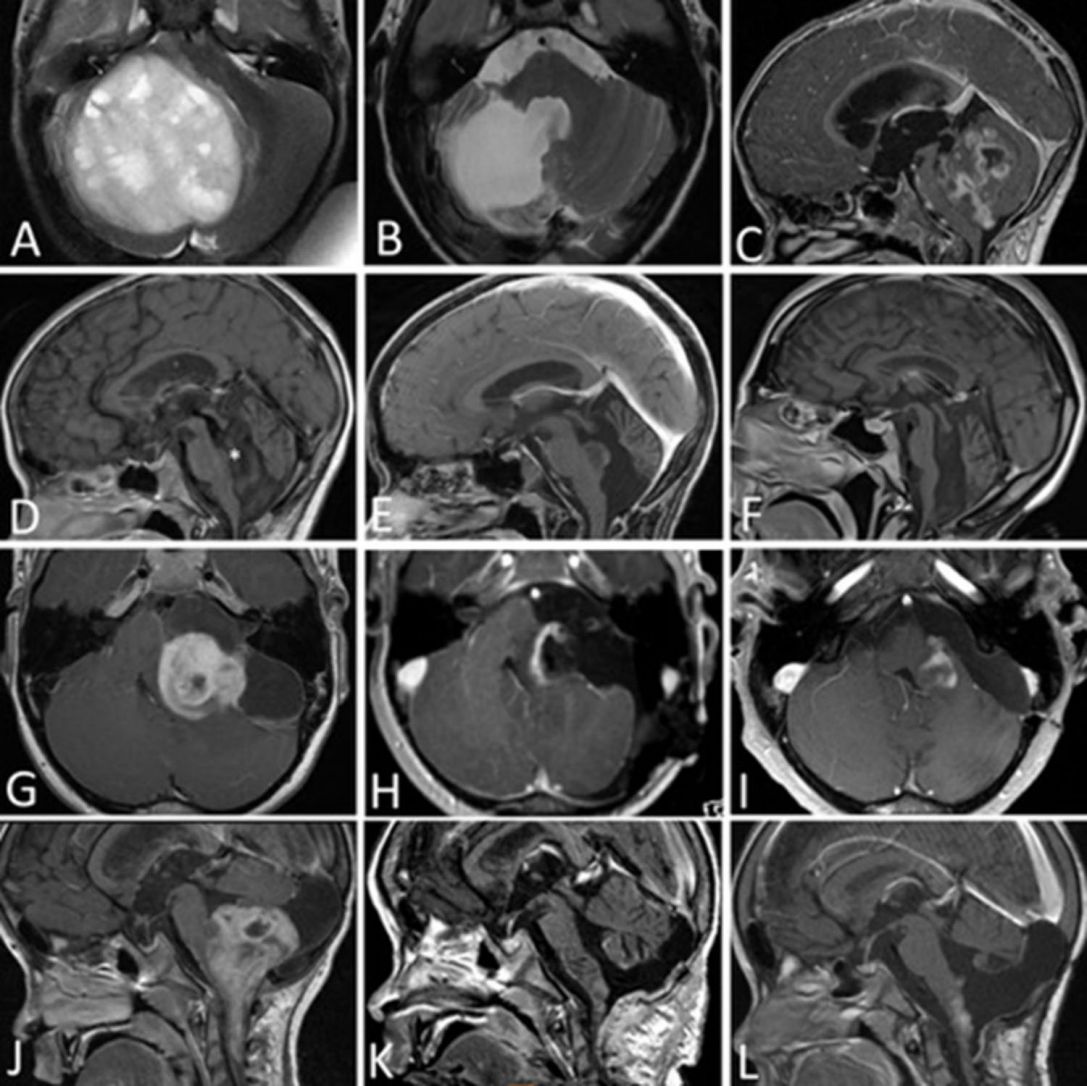
One example of BS-pLGG per group according to location. A, B *Cerebellopeduncular pLGG* in a 2-year-old boy prior to surgery (A) and with 10-year FU after GTR without recurrence (B). C–F *4th ventricular pLGG* with brain stem invasion in a 7-year-old girl prior to surgery (C). After surgery, there is minimal residual tumor left at the infiltration zone of the 4th ventricular floor (D). Five years later, the progressive residual underwent GTR and no recurrence was seen after another 5 years FU (F). G–I *Pontine pLGG* exophytic into the CPA in a 7-year-old boy presenting with high-grade facial pareses and deafness on the left (G). After STR, there is the intentionally left residual tumor alongside the facial nerve (H), which recovered to normal function. The slowly growing residual underwent another STR after 5 years (I); however, the residual showed immediate progression and is currently stable under Vinblastin treatment. J–L *Medulla oblongata pLGG* with intrinsic and exophytic growth pattern in a 4-year-old boy (J) prior to surgery and after STR (K). The residual tumor showed mild progression 2 years (L), and chemotherapy was recommended in his distant home institution. The boy was lost to FU thereafter

Patients were stratified according to anatomic location into four cohorts: Group 1, Cerebello-peduncular tumors (*n* = 14); Group 2, 4th ventricle tumors with brain stem invasion (*n* = 14); Group 3, tumors with pontine and CPA involvement (*n* = 9), and Group 4, tumors with primary medulla oblongata manifestation (*n* = 15). The examples of one tumor per group are shown in Fig. 1.

*Molecular genetic profiling* was missing in 5 cases due to operations performed before 2010 or due to the decline of molecular diagnostics by parents/legal guardians.

In the remaining 47 cases, we found 24 KIAA1549::BRAF fusions, in one of them an additional PALB2-frameshift mutation (this patient with cerebello-peduncular tumor underwent GTR and stayed free of disease during follow-up). In 13 cases, no genetic alterations were seen; in 4 patients, BRAF-V600E mutations; in 2 patients, segmental loss of chr. 9 p inclusive CDKN2A/B gene; and in one each, a BRAF-GNAI fusion gene a, an ATM-Stopgain-Mutation, a 3-base insertion of Exon 15 of BRAF gene, and a PMS-2 CREBBP gene variant.

Four patients harbored an NF1 mutation. No other molecular genetic alterations except NF1 gene mutation were detected in this group.

In the most common genetic alteration, the KIAA1549::BRAF fusion was present in 64% of cerebello-peduncular tumors, in 57% of the 4th-ventricle group, in 33% of pontine/CPA tumors, and in 26.6% of tumors of the medulla oblongata.

### Primary surgical strategies and post-interventional tumor behavior (Table 1, Fig. 1)

Robotic biopsy was performed in 2/52 cases, one patient was thereafter treated with Trametinib and showed SD at the last follow-up, and the other patient underwent Vinblastin treatment and was lost to long-term FU.

A total of 2/52 patients underwent chemotherapy as primary treatment in other institutions. Both showed tumor progression and received surgical intervention at our service. For further analysis, they were included in the primary surgical series and fell into the partial resection group (PR).

Thus, 50/52 patients underwent surgery as the first intervention (2/52 had neoadjuvant chemotherapy before their first surgery). Among these, 9/50 experienced GTR (18%). Of the 41 patients with incomplete resection (82%), STR was achieved in 18/41 (46%) and PR in 23/41 (54%).

The surgical approach was chosen according to the tumor location and extension. The best-suited approach was selected to enable ideal exposure, use safe entry zones, and avoid crossing displaced cranial nerves. All cases were approached either by a median suboccipital approach or with unilateral extended exposure in the case of a subtonsillar approach to lateral-inferiorly extending tumors (below the exit zone of CN VII/VIII). Only the telovelar not the transvermian approach to the 4th ventricle was applied. For the upper lateral brain stem and upper CPA extension, a standard retrosigmoid approach was used, combined if necessary with a lateral supracerebellar approach. For all midline approaches, the prone position was used. For retrosigmoid approaches, the standard position was supine with the head turned to the contralateral side (modified Jannetta position), and a semi-sitting (lounging) position was used in selected patients.

All cases were operated on with extended intra-operative electrophysiological monitoring, including SEP and MEP, BAEP, MEP to all motor nerves (III, V–VII, IX–XII), free-running EMG, and direct stimulation of the pyramidal tract in the cerebral peduncles or motor cranial nerves. All involved functions were monitored if possible. Functionality was given priority over the extent of resection, and resection was stopped if MEP deteriorated to about 30% of initial values without fast recovery within 5 min.

### Progression/stability after first surgical resection and following treatment (Table 1, Fig. 2, Supplementary information 1)

After surgical resection, 26/50 patients (52%) showed no recurrence after GTR or no further progression after incomplete resection, remaining stable during follow-up observation. This included all 9 patients with GTR (100%), 10/18 patients with STR (56%), and 9/23 patients with PR (39%).

The remaining 8/18 patients with STR (44%) showed progression, indicating the need for a second intervention. Two of the ten patients with long-term stability showed some radiological progression at the last follow-up without clinical symptoms and were further observed.

A total of 14/23 patients with PR (61%) showed progression after the first surgery during follow-up and eventually required further treatment. Overall, 22/41 patients (48%) with incomplete resection showed tumor progression, necessitating further treatment. Of these, 15/22 patients (68%) underwent a second surgery, 1/22 received targeted therapy (Trametinib), and 6/22 (27%) were treated primarily with chemotherapy. In 2/6 cases, chemotherapy failed to induce long-term stability, leading to a second surgery.

Among the 17 patients who underwent a second surgery, 8 (47%) remained stable. Of the 9 patients with further progression, 5 had a third surgery, and 2/5 (40%) remained stable thereafter. One patient underwent four surgeries and still had progressive disease at the last follow-up.

Additionally, two patients were diagnosed with spinal metastasis and underwent three spinal tumor resections.

**Fig. 2:**
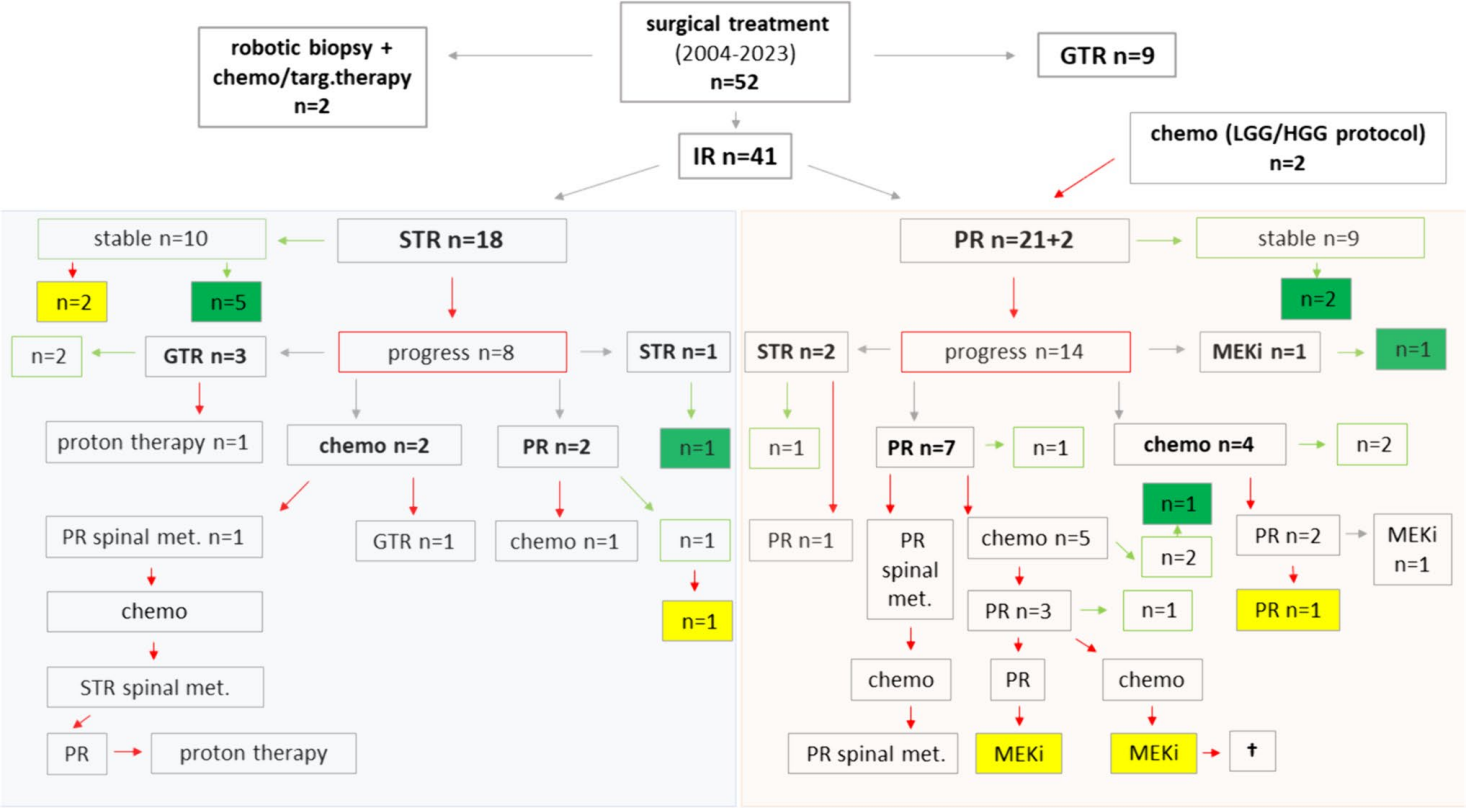
This figure summarizes the therapy sequelae of patients who underwent surgical resection/robotic biopsy in our institution between 2006 and 2020. Abbreviations: n, number of patients; chemo, chemotherapy; GTR, gross total resection; IR, incomplete resection; STR, subtotal resection; PR, partial resection; MEKi, MEK-inhibitor; green arrow, no progress; red arrow, progress; yellow highlight, progress at last follow-up (according to RANO 2.0); green highlight, remission at last follow-up (minor or partial response (*MR*, *PR*) according to RANO 2.0) [14]

Table 1, Fig. 2, and Supplementary Information 1 provide details regarding the overall treatment options and the treatment successes and failures.

**Table 1:** This table shows the outcome (progression, complete response (*CR*), or stable disease (*SD*)) of patients after the first, second, and third resection of intracranial manifestations. Fifty-two percent of patients were free of disease during follow-up or in *SD* after first surgery. Secondary surgery after initial subtotal resection (STR) yielded treatment response in 3/6 patients (50%). After partial resection (PR), a second surgical intervention was less successful, and only 2/9 patients (22%) led to treatment response. Stability after a 3rd resection was still induced in 2/5 cases (40%). *GTR* gross total resection, *STR* subtotal resection, *PR* partial resection, *na* not applicable, *No* number, *pts* patients

**Resection rate** Primary surgery (+ 2 with surgery after primary chemotherapy)	**GTR** 100% removed	**STR** > 90% removed	**PR** < 90% removed
**No. of pts**	9/50 (18%)	18/50 (36%)	23/50 (46%)
**Stable disease**	9 (100%)	8/18 (44%)	9/23 (39%)
**Progression** Demanding treatment	0	8/10 (80%)	14/14 (100%)
**Primary second surgery**	0	6/8 (75%)	9/14 (64%)
GTR		3/6 (50%)	0
STR		1/6 (17%)	2/9 (22%)
PR		2/6 (33%)	7/9 (78%)
*CR/SD* after primary 2nd surgery		3/6 (50%)	2/9 (22%)
*CR/SD* after secondary 2nd surgery		1/1	1/2
**Third surgery**		0	5
Stability after 3rd		na	2/5(40%)

The 24 patients (48%) that showed progression demanding treatment underwent altogether 23 further intracranial surgical interventions, 15 chemotherapy applications, 4 targeted treatments with MEKi, and 2 proton radiation treatments. Two patients were diagnosed with spinal metastasis and underwent three spinal operations. One patient developed cranial and spinal meningeosis.

### Neurological outcome, overall survival (OS), progression free survival (PFS), and complications

Direct postoperatively 31 patients (62%) showed moderate to severe neurological deficits (cerebellar mutism *n* = 4, cranial nerve dysfunction *n* = 16, central sleep apnea *n* = 4, ataxia *n* = 7), of which, 27 patients recovered in 12 months; at 1-year follow-up, only 4 patients presented with moderate neurological deficits. At the last follow-up, 3 patients (6%) had moderate neurological deficits that allowed schooling but affected everyday life, 26 patients (52%) had mild neurological deficits not affecting activities, and 18 patients (36%) had no neurological deficits (see Table 1 Supplementary material).

OS during the observational period was 98.1%, and the mortality was 1.9% (1/52 patients).

The mean PFS of the overall cohort was 34.07 months.

Complications after surgery occurred in 4 cases (7.6%) (wound revision surgery, 1 patient; epidural bleeding, 2 patients, (one with first diagnosis of factor 13 deficiency); pneumocephalus, 1 patient).

### PFS of patients treated exclusively surgically

The mean PFS after the first operation was 44.2 months. In this analysis, we included only those 43 patients who underwent surgery as primary therapy without chemo/radiotherapy direct pre-or postoperatively or without chemo/radio/targeted therapy after robotic biopsy. Patients not included — altogether 9—: robotic biopsy + chemo/targeted therapy *n* = 2, chemo as primary therapy or direct pre-or postoperatively *n* = 7).

Seven patients with progression underwent second surgery without ever receiving radio/chemo/targeted therapy thereafter. In this group, the mean PFS after 2nd surgery was 38.3 months. One of these patients showed progress, underwent 3rd surgery, and had 130.5 months PFS.

### Outcome at last follow-up

At the last follow-up, the outcome of 50 patients was analyzed, since 2 patients were lost for follow-up longer than 12 months after surgery. The outcome was evaluated using the RANO 2.0: Update to the Response Assessment in Neuro-Oncology Criteria for Low-Grade Gliomas scheme [14] (see Fig. 1).

Sixty-eight percent of patients (*n* = 34) were either in *SD* after GTR (*n* = 11) or had *SD* after IR (*n* = 23).

Twenty percent (*n* = 10) showed *tumor regression* in the volumetric analysis. Tumor regression was defined as a minor response (*MR*) in case of a minimum 40% decrease in tumor volume and as a partial response-(*PR*) if a ≥ 65% decrease in tumor volume was achieved. No complete response (*CR*) was observed. Five patients showed minor remission — two patients after STR, one after two STRs, one after PR, and one patient after PR and MEKi. Partial remission was seen in 5 cases, in three after STR, in one after PR, and in one patient after PR plus PR plus chemotherapy. Thus, the majority (8/10) of minor or partial remissions occurred after surgical treatment only.

Twelve percent (*n* = 6) showed *progression* of disease (*PD*, minimum of 40% increase in volume confirmed on two consecutive images). Three tumors with progression at the last follow-up were located in medulla oblongata with histological diagnosis of PCA and underwent before 3–4 PR and were also treated with chemo/targeted therapy.

The only mortality was a female NF1 patient with a medulla oblongata pilocytic astrocytoma. She died at age 22 after 12 years of treatment, which included multiple resections, chemotherapy with vincristin/carboplatin and vinblastin, and MEKi treatment that eventually led to resistance. She passed away from respiratory insufficiency just before scheduled radiation therapy. Despite her illness, she maintained a good quality of life, finishing school, completing an apprenticeship, and working as an accountant for 2 years (see Table 1 and Fig. 1).

The other three patients who had *PD* at the last follow-up underwent only surgical interventions: two of them had a single STR, and one underwent after PR a second surgery. *PD* occurred at 1 year, 3 years, and 5 years follow-up. These patients were still observed as they were asymptomatic, and tumor volume was still not significant—(0.469 cm^3^, 2.97 cm^3^, 3.55 cm^3^).

At the end of a mean follow-up period of 6.4 years, 25/50 patients (50%) were tumor-free or showed partial remission of residual tumors, and 19/50 (38%) had stable disease (*SD*) with a non-progressing residual tumor. Consequently, 44/50 patients (88%) had successful treatment of a BS-pLGG. Only 6/50 (12%) showed progression; one died, three were under observation only, and one had moderate progression under MEKi.

### Characteristics of BS-pLGG according to the location of tumor (details see Supplementary information 1)

**Fig. 3:**
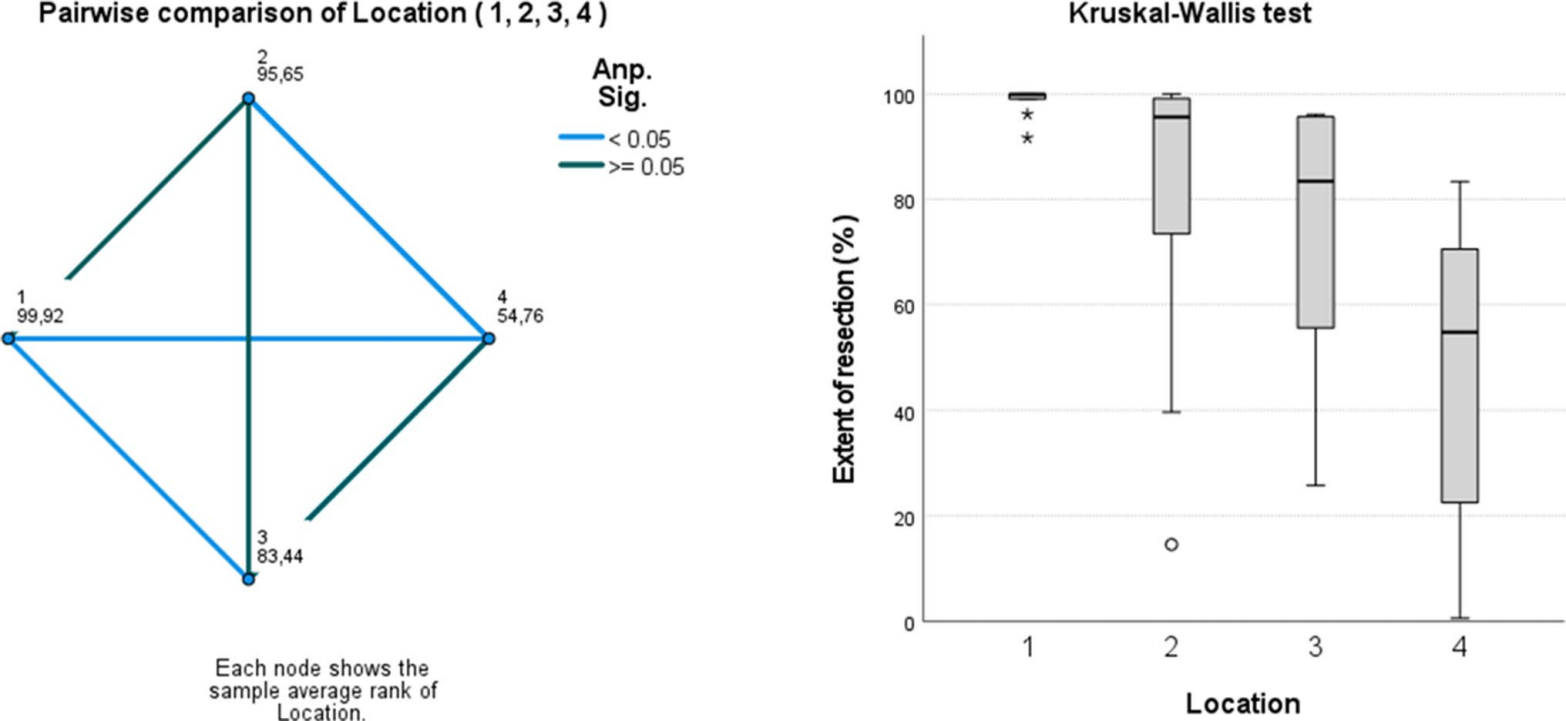
Location of tumor influenced extent of resection (EoR, % of pre-operative tumor volume removed). The highest EoR was achieved in Group 1, cerebellar-peduncular tumors followed by Group 2, 4th ventricle tumors, and Group 3, pontine/CPA tumors. The lowest EoR was achieved in Group 4 medulla oblongata tumors. (Kruskal–Wallis test, *p* = 0.004)

The location of tumor seemed to significantly influence the extent of resection. The extent of resection was expressed in the percent of removed volume compared to the initial volume (%) and calculated based on preoperative and postoperative 3-dimensional volumetric analysis. The extent of resection was in part translated into different PFS times within the four groups (see Fig. 3 and Table 2).

**Table 2:** Extent of resection of pre-operative tumor volume at first surgery, progression-free survival (PFS) in months after first surgical intervention, and oncological outcome at last FU in the four groups defined by tumor location: Group 1, cerebellar-peduncular tumors; Group 2, 4th ventricular tumors; Group 3, pontine/CPA tumors; Group 4, medulla oblongata tumors. *GTR* gross total resection, *IR* incomplete resection, *PFS* progression free survival, *FU* follow up, *SD* stable disease, *PD* progressive disease

Group	Group 1 *n* = 14	Group 2 *n* = 14	Group 3 *n* = 9	Group 4 *n* = 15
Pilocytic astrocytoma	100%	93%	89%	67%
Ganglioneural tumors	0%	7%	11%	27%
KIAA1549_BRAF fusion	64%	66%	33%	31%
BRAF V600E mutation	7%	7%	0%	15%
Other BRAF alteration	0%	7%	11%	0%
No genetic alteration	21%	14%	44%	31%
CDKN2A deletions	0%	0%	0%	13%
GTR at last FU (%)	50%	14%	0%	0%
Extent of resection in IR cases after first surgery *(% of tumor volume, mean* ± *SD)*	98.9%	80.8%	73.3%	48.1%
Biopsy and systemic treatment	0%	0%	11%	7%
PFS *(months, mean* ± *SD)*	50.8 ± 15.6	47.9 ± 14.1	12.5 ± 3.5	38.3 ± 11.8
Further therapeutic interventions (%)	0%	50%	66%	64%
Free of tumor at last FU (%)	50%	14%	0%	0%
*SD*/regression at last FU (%)	36%	86%	89%	71%
*PD* at last FU* under observation (%)*	14%	0%	0%	0%
Disease controlled at last FU (%)	100%	100%	89%	71%
*PD* at last FU* requiring treatment (%)*	0%	0%	11%	22%
Mortality	0%	0%	0%	7%
Neurological deficits interfering with daily life at last FU (%)	0%	0%	12%	14%

The four groups showed the following characteristics, for more details see Table 2 and Fig. 1.

- Group 1: Cerebello-peduncular BS-pLGG, *n* = 14 — the most favorable group. These tumors were located predominantly within the cerebellar peduncle and the adjacent cerebellum with minor involvement of medulla oblongata, pons, or CPA.

Fifty percent of patients underwent GTR and 50% STR. At the last follow-up, all GTR patients remained tumor-free. In the STR group, 2 had *PR* (14%), 1 had *MR* (7%), 2 had SD (14%), and 2 had *PD* (14%). The latter had small tumors and were observed without additional therapy. The mean PFS was 50.8 months, and the mean follow-up was 5.45 years.

Immediate postoperatively, 7 patients (50%) had moderate to severe neurological deficits (including cerebellar mutism and ataxia), but long-term follow-up showed no significant deficits affecting daily life in all.

- Group 2: BS-pLGG of IV-th ventricle, *n* = 14 — the second most favorable group. These tumors occupied predominantly the 4thventricle with various extents of infiltration of the brainstem/cerebellar peduncle.

All patients underwent first-line surgical treatment: 2 GTR, 6 STR, and 6 PR. Half (*n* = 7) showed no recurrence or progression (2 GTR, 2 STR, 2 PR). The other half (*n* = 7) showed progression, with five undergoing second surgery, one receiving chemotherapy, and one MEKi therapy. After a second surgery, one patient achieved *SD* with proton therapy. At the last FU, no patients had progression: 2 were tumor-free (*SD* after GTR), 8 had *SD* after IR, 2 had *MR*, and 2 had *PR*. The mean PFS was 47.9 months, with a mean FU of 7.6 years.

Immediate postoperatively, 10 patients (71%) had moderate to severe neurological deficits (cerebellar mutism *n* = 3, central sleep apnea *n* = 1, cranial nerve dysfunction *n* = 3, ataxia *n* = 4). At the last follow-up, 8 had no neurological deficits and 6 had mild deficits.

- Group 3: BS-pLGG with predominantly pontine and cerebellopontine angle (CPA) involvement, (*n* = 9) — the complex group with an overall good outcome.

In this group, *3 patients had tumors with pedunculo-pontine location with CPA involvement, 2 patients with pedunculo-pontine location without CPA involvement, and 4 patients with pedunculo-pontine-medullary location.*

One patient underwent a robotic biopsy and first-line MEKi treatment, showing SD. Four patients had PR as first-line treatment, with 2 showing *SD* and 2 *PD*. Four patients had STR, all showing *PD*.

All six patients with *PD* after the first intervention needed further treatment. Two received chemotherapy, both showing *PD*. Four had second surgeries, with one achieving *MR* and three showing further *PD*. A third intervention was needed for four patients: one achieved *SD* with chemotherapy, two had *PD* after the second surgery, and one had *SD* after a third resection. Four or more interventions, including proton therapy, achieved *SD* in two patients. At last FU, one patient was in *MR*, seven (77.7%) were in *SD*, and one showed *PD*. The mean PFS was 12.5 months, and the mean FU was 5.1 years.

One patient was lost to FU. At the end of FU, seven patients had mild to no deficits affecting daily life and all were enrolled in school.

- Group 4: BS-pLGG predominantly within the medulla oblongata, *n* = 15, the least favorable group.

This group had the most complicated disease course. One patient (6%) underwent a robotic biopsy and chemotherapy and was lost to follow-up. Due to electrophysiological constraints, only PR could be reached for the remaining 14/15 patients.

After PR, 5/14 (36%) showed sustained stability (4 *SD* and 1 *MR*), while 9 showed progression (*PD*). Four patients had chemotherapy as a second intervention, with 3 achieving *SD*. Five had a second PR, all showing *PD*. As the third intervention, four patients had chemotherapy and two had PR; only one showed partial remission (*PR*), while five had further *PD*, requiring four or more interventions.

At the last follow-up, ten patients (71%) were stable (8 SD, 2 remission), three remained in *PD*, and one died. Nine patients (60%) received chemotherapy, and three (20%) received MEKi during observation. The mean PFS after the first intervention was 38.3 months, with a mean FU of 7.8 years.

Two patients had a segmental loss of chr. 9p CDKN2A/B gene and showed a particularly complicated course, with one dying and the other showing sustained progression despite long multiple treatments including MEKI. Postoperatively, ten patients had moderate to severe neurological deficits (swallowing problems, ataxia, cranial nerve dysfunction, central sleep apnea). Most recovered, with only two having long-term moderate deficits affecting daily life but allowing schooling.

### Tumor growth velocity cm^3^/months

A total of 36 out of 52 met the inclusion criteria for TGV calculations. Patients with GTR without relapse were excluded. Among the remaining patients, 10 had primary STR and 14 had PR. Eight underwent a second surgery, three had a third surgery, and one had a fourth surgery. The Shapiro–Wilk test indicated a non-normal distribution of total growth volume (TGV) data (*p* < 0.05).

We compared TGV in patients who underwent solely surgical resection (*n* = 25; STR *n* = 10, PR *n* = 9, multiple operations *n* = 6) with those who also received chemo/radiotherapy or targeted therapy (*n* = 11). Patients treated surgically only had lower cumulative TGV compared to those receiving additional therapies (Mann–Whitney test, *n* = 36; Mann–Whitney *U* = 233, *z* = 3.28, *p* = 0.001).

The purely surgical group (25 patients) showed a trend toward decreased mean TGV after successive operations (mean TGV after 1st operation = 0.072 cm^3^/month, after 2nd operation = 0.021 cm^3^/month, after 3rd operation = 0.0003 cm^3^/month), though this did not reach statistical significance.

When comparing patients with disease progression who underwent only surgical resection without neoadjuvant treatment (10 patients; 5 STR, 5 PR), STR patients exhibited significantly lower TGV compared to PR patients (Kruskal–Wallis test, *n* = 5 STR, *n* = 5 PR, df = 1, Test Statistic = 4.81, *p* = 0.028).

**Fig. 4:**
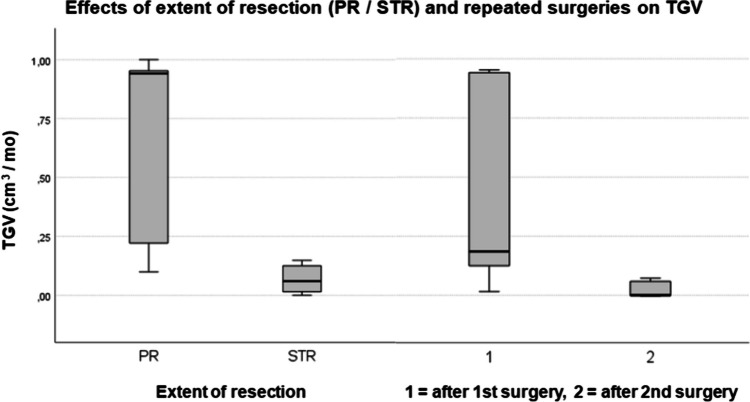
This figure shows the effects of the extent of resection (PR/STR) and repeated surgeries on tumor growth velocity (TGV cm3/month). Patients after *PR* surgery who had *PD* showed higher TGV than patients following *STR* with *PD* (Kruskal–Wallis test, *n* = 5 STR, *n* = 5 PR, df = 1, Test Statistic = 4.81, *p* = 0.028). Distribution of TGV in patients who underwent 2 resective surgeries without neoadjuvant treatment. 1: TGV after the first surgery, 2: TGV after the second surgery, Wilcoxon test showed a significant difference between the two groups (*z* =  − 2.2, *p* = 0.028), *n* = 6

Additionally, a pairwise comparison of patients undergoing first and second surgical treatments without neoadjuvant therapy showed significantly decreased TGV after the second operation (TGV = 0.021 cm^3^) compared to after the first surgical resection (mean TGV = 0.40 cm^3^) (Wilcoxon test, *n* = 6, *z* =  − 2.2, *p* = 0.028) (Fig. 4).

### Molecular genetic alterations

**Fig. 5:**
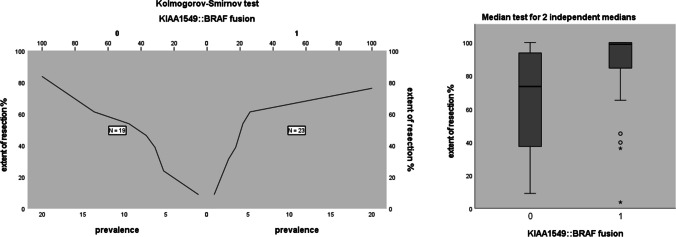
Median test for 2 independent medians showed a significant difference of the extent of resection comparing patients with and without KIAA1549::BRAF fusion. 0 = no KIAA1549-BRAF fusion, 1 = KIAA1549::BRAF fusion

KIA1549::BRAF fusion was associated with the extent of resection. In this analysis, we included all patients with available data (pre- and postoperative scans after first surgery independent of resection rate and molecular genetic analysis) altogether 42 patients. Twenty-three patients had KIA1549::BRAF fusion and 19 did not (among these patients 3 had BRAF-V600E mutation). We found a significantly higher extent of resection in patients with KIA1549::BRAF fusions compared to patients without (Kolmogorov–Smirnov test, *p* = 0.21, median test for 2 independent medians, *p* = 0.013), see Fig. 5. Most of the STR and GTR was achieved in the group of patients with KIA1549::BRAF fusions, *p* = 0.021.

## Discussion

This institutional study on BS-pLGG in 52 children and adolescents analyzes the role of surgery stratified to different groups of origin and brain stem involvement. Most patients were diagnosed and treated during early childhood. Only two patients underwent initial “biopsy only” followed by systemic treatment. The analysis includes 50 patients who underwent open surgical intervention, with a mean FU of over 6 years (minimum 3 years).

Resection was always guided by intraoperative ultrasound similar to the algorithm described recently by Frassanito et al. [15]. Additionally, intraoperative MRI (iMRI) was used in selected cases like in complex pontine tumors with CPA extension as described previously by our group [16].

Surgical strategy adhered strictly to intraoperative electrophysiological monitoring guidelines, including MEPs, AEPs, and EMG, aiming for long-term function preservation and achieving “minimal safe residual” tumor volume as defined previously [6]. A decrease of MEPs to a maximum of 30% of the initial value was defined as a “stop sign,” and resection was continued only in case of recovery of MEPs. In free-running EMG severe A trains were a stop sign, for AEPs, this was a significant drop to 50%. Post-surgery, 60% of patients experienced new neurological deficits, mostly mild to moderate, with 87% of these recovering within a year or achieving functional levels compatible with daily activities. At follow-up, 6% had moderate deficits, 52% had mild deficits not impacting daily activities, and 36% had normal neurological exams. No patients required tracheostomy.

With additional treatment such as chemotherapy, MEK-inhibition, or radiation, 50% achieved *SD* or *PR*, and 38% remained stable with non-growing tumor remnants (*SD*). Overall disease control rate was 88%, with 10% showing progression, though most of these patients were under observation without active treatment. Unfortunately, one patient died from therapy-resistant disease in the medulla oblongata.

## Influence of extent of resection and location

We recently published a quantitative analysis of residual tumor growth dynamics in a diverse cohort of pLGG patients treated at our institution. Our findings demonstrated a volume-dependent reduction in post-operative growth rates, establishing the concept of a “safe minimum residual” volume [6]. Below a threshold of 2 ml residual volume, the risk of further tumor growth significantly decreased (AUROC = 0.88). We observed significant growth deceleration in supratentorial hemispheric and infratentorial tumors compared to supratentorial midline gliomas [6].

This study combines existing volumetric data with new cases to analyze the subgroup of BS-pLGG in greater detail.

Similar to recent studies on BS-pLGG, including one with 62 patients undergoing surgical resection [2], and another analyzing 116 children from the German SIOP-LGG 2004 cohort [1], we observed excellent OS of 98% and PFS dependent on the extent of resection and tumor location (see Table 2). In the cerebellar-peduncular and 4th ventricle Groups 1 and 2, we achieved the highest rates of GTR, with the longest PFS of 50.8 and 47.9 months respectively, and 100% disease control at follow-up (tumor-free or *SD*). Patients with STR had a 44% progression rate compared to 61% in the PR group, with all PR patients receiving further treatment. Two STR patients showed minimal progression and were observed without active treatment.

In the series by Rady et al. [2], 12 patients underwent biopsy only, while 50 underwent open surgery, categorized differently from our stratification. They reported higher GTR rates (40%) compared to our cohort (18%), possibly due to a higher proportion of tumors involving the upper and lower peduncles (70% vs. 30% in our classification). There may be an overlap between their peduncular group and our 4th ventricle tumors. However, comparability regarding residual tumor behavior is limited due to their different definitions of STR and PR (> 95% as near total and < 95% as subtotal). Additionally, they estimated rather than quantitatively measured residual tumor volume as in our protocol. We calculated PFS in months, while Rady et al. reported percentages at 3 or 5 years, which may explain their higher rates of progression-free patients. Nevertheless, both studies suggest a positive association between larger resection extent and lower tumor progression rates.

This principle is similarly observed in the larger SIOP LGG 2004 cohort of 116 patients from 71 centers, including many low-volume centers. They used a comparable classification for the extent of resection, although not based on volumetric analysis. Nonetheless, the authors found a significant correlation between event-free survival and the extent of resection (GTR, STR, and PR) [1].

Striving for minimal safe residual (maximal safe resection) not only improves PFS but can also reduce long-term chronic health burdens caused by delayed effects of chemotherapy and radiotherapy on posterior fossa structures. Gojo and Preusser [8] [7] reported cumulative rates of chronic health conditions in 2501 pLGG survivors treated with radiotherapy, surgery, or chemotherapy as primary treatment, with a median follow-up of 24 years. They found that radiotherapy had the highest rates (24.5%), followed by chemotherapy (14%), while surgery-only patients had the lowest rates (8.3%). They also noted a shift in treatment trends for BS-pLGG from radiotherapy in the 1970s–1990s towards surgical resection alone [7, 17].

This requires that surgery has minimal impact on long-term neurological function. Surgery was the primary treatment in nearly all cases (96%; 50/52 cases) in our cohort and was considered first-line for secondary tumor progression. When complete resection was not feasible due to functional limitations, we aimed for further tumor volume reduction in subsequent surgeries (minimal safe residual concept). Only 1 case (2%) required intracranial radiation therapy, and chemotherapy was used in ten patients (20%). Fortunately, our monitoring and imaging-based approach resulted in excellent long-term neurological outcomes: no initial tracheotomies or gastric feeding tubes were needed, severe permanent neurological deficits were absent, and only three patients (6%) experienced moderate deficits affecting daily life.

In the German SIOPe-LGG 2004 cohort [1], with a mean follow-up of 6.8 years, fewer patients underwent primary surgical resection (63%, 73/116) compared to our cohort (96%), resulting in more upfront chemotherapy use. Following primary surgery, our cohort had a higher rate of secondary surgeries (68%, 15/22) compared to SIOPe-LGG 2004 (4%, 2/53). In patients with initial STR, we achieved GTR in 50% (3/6) at the second surgery, and three more achieved STR or PR. In contrast, 49% of SIOPe-LGG 2004 patients received non-surgical adjuvant treatments: 33% chemotherapy and 16% radiation therapy. Due to more frequent surgery in our cohort, only 20% ever received chemotherapy. Similar rates of *SD* were achieved in both cohorts, but SIOPe-LGG 2004 reported 16% with significant long-term neurological deficits and 9% deaths from disease or treatment complications. Despite higher surgical rates and less chemotherapy and targeted treatment in our cohort, we achieved a more favorable neurological outcome (6% with moderate deficits), one fatality, and less impact on quality of life by avoiding chemotherapy or radiation.

The differences in neurological outcomes may stem from differences in tumor locations (details not provided in Holzapfel et al.) [1], or from our specialized pediatric brain tumor center’s higher case volume and expertise in using neuromonitoring. Holzapfel et al. [1] suggested that concentrating surgical interventions at experienced, high-volume centers could enhance neurological outcomes. Our findings appear to endorse this approach.

However, location also appears to influence the effectiveness of second or third surgeries in cases of progression. In the challenging Group 4 of medullary tumors, six patients underwent a second surgery upon progression, resulting in only PR in all cases. Subsequently, all six patients experienced further progression and required chemotherapy. This suggests, that a second surgical intervention following initial surgery for progression should not be considered in medullary tumors where achieving GTR or STR is highly unlikely.

Furthermore, we observed that patients treated solely with surgical resection had lower residual TGV compared to those who also received chemotherapy, targeted therapy, or radiation. This likely reflects the aggressive nature of tumors that did not respond to surgery alone, necessitating additional treatments. Ultimately, only 6% of patients (*n* = 3) showed progression at the last follow-up. This suggests, that patients who were unable to undergo complete or subtotal surgical resection and continued to experience tumor growth after initial or subsequent surgeries could still achieve *SD*, excellent OS, and good PFS through interdisciplinary management and multilevel therapy, as described by other research groups as well [2, 4, 18].

Distribution of histological entities showed approximate conformity to a previously published population-based cohort of BS-pLGG [1].

In recent years, the incremental unraveling of the molecular landscape of pediatric CNS neoplasms has not only led to a more profound understanding of their biological foundations but also fostered increasingly advanced stratification and the implementation of novel targeted therapies [19–21]. In pLGG, a near-ubiquitous upregulation of the RAS/MAPK pathway, frequently involving single nuclear variants (SNV) and fusion genes of BRAF, FGFR1/2 and NF1 could be discovered [22–25]. While recent molecular analyses found significantly enriched molecular driver alterations at distinct tumor sites such as KIAA1549::BRAF fusions in tumors of the posterior fossa or BRAF-V600E mutations in tumors involving the cerebral hemispheres, no particular hallmark alteration has been described in BS-pLGG [22, 26].

Within our cohort, a high frequency of KIAA1549::BRAF fusions was observed due to the predominance of PCAs, as this particular driver alteration shows a high prevalence in these tumors [22, 26]. Notably, KIAA1549::BRAF fusion genes were predominantly found in tumors of Group 1 (cerebello.peduncular tumors) and Group 2 (4th ventricle origin) which showed minor involvement of the medulla or pons therefore resulting in higher resection rates and a superior outcome in BS-pLGG bearing this particular driver alteration. This observation, however, has to be evaluated in larger, population-based cohorts.

Notably, a relatively high frequency of BRAF-V600E mutations was detected among gangliogliomas (3/5), showing accordance with a previously published series describing a significant enrichment of this SNV in pediatric brainstem gangliogliomas [27]. Data from previous integrated molecular and clinical analyses indicate an inferior outcome and an increased risk of malignant transformation to secondary HGG of tumors bearing this particular driver alteration, particularly with co-occurring CKDN2A/B co-deletions [28–30]. These particular patients, however, may prospectively significantly benefit from advanced targeted therapies. Recently, a combinational treatment regimen involving MEK inhibitor trametinib and BRAF inhibitor dabrafenib has shown significantly higher response rates and superior tolerability as compared to the standard-of-care chemotherapy regimen involving carboplatin and vincristine in BRAF V600E positive pLGG, therefore currently being considered the first-line non-surgical treatment approach in these patients [31]. Further molecular therapies including MEK, 1st generation BRAF, FGFR, and RAF inhibitors have previously shown promising results in phase I/II studies and are prospectively under evaluation in randomized controlled studies, potentially offering promising complementary treatment options in BS-pLGG patients showing progressive disease due to limited resectability [32–36].

## Role of intraoperative MRI

The beneficial impact of intraoperative MRI (iMRI) on pediatric low-grade glioma surgery has been supported by single-center and multicenter studies [16, 37] and confirmed by a meta-analysis showing the improved extent of resection (EoR) without adverse effects on neurological outcomes typical in pediatric neuro-oncological surgery [38].

In our cohort of pBS-LGG, iMRI was utilized in 10 procedures. It confirmed gross total resection (GTR) in 2 cases (both reoperations in Group 2 tumors). In the other 8 cases, iMRI revealed the precise extent and location of residual tumors, which could be partially reduced but not completely removed in 6 cases: 2 cases each in Group 2, Group 3, and Group 4. In all 6 patients, resection was limited by electrophysiological constraints. In 2 additional cases (Group 4 and Group 2), iMRI was performed but no further resection was attempted due to the critical eloquence of the area. Thus, iMRI contributed to increasing EoR in 60% of cases within the infiltration zone until electrophysiological limits were reached.

Following the implementation of a new intraoperative ultrasound system in 2018 with improved imaging quality, the additional use of iMRI was deemed of no further value thereafter, also given the dominance of the electrophysiological guidance and constraints over visible residual tumors.

## Limitations

One of the limitations of our study is its retrospective setting and variable length of FU periods that can distort results when comparing PFSs. In spite of these limitations, our results resembled similar tendencies as those of larger cohorts. Further prospectively designed studies could show to what extent these tendencies observed in our single institutional cohort apply.

## Supplementary Information

Below is the link to the electronic supplementary material.

ESM 1 The Supplementary information 1 table shows the main characteristics of the 4 cohorts in detail based on location groups and contains data on number of patients (pts)., gender, resection rates and sequelae of treatment, reoperations, use of iMRI (number of surgeries), hydrocephalus, chemotherapy, targeted therapy, radiotherapy, molecular genetic alterations, histology, symptoms at diagnosis, clinical presentation, early-and long-term neurological outcome, progression free survival (months), rehabilitation.(XLSX 10.6 KB)

## Data Availability

No datasets were generated or analysed during the current study.

## Abbreviations

CPA: Cerebellopontine angle
*CR*: Complete response
EoR: Extent of resection
FU: Follow-up
GGL: Ganglioglioma
GTR: Gross-total resection
IR: Incomplete resection
*MR*: Minor response
*PR*: Partial response
PR: Partial resection
PCA: Pilocytic astrocytoma
pLGG: Pediatric low-grade glioma
BS-pLGG: Pediatric low-grade glioma affecting the brain stem
RGNT: Rosette-forming-glioneural-tumor
STR: Subtotal resection
TGV: Tumor growth velocity
QoL: Quality of life
